# Modeling Post-Implantation Mammalian Embryogenesis Using Advanced *In Vitro* Systems: From Mice to Humans

**DOI:** 10.3390/ijms27020900

**Published:** 2026-01-16

**Authors:** Dongsong Liu, Yiwei Zhang, Tianyao He

**Affiliations:** 1 State Key Laboratory of Reproductive Regulation and Breeding of Grassland Livestock, School of Life Sciences, Inner Mongolia University, Hohhot 010070, China; 2 Key Laboratory of Animal Cellular and Genetics Engineering of Heilongjiang Province, College of Life Science, Northeast Agricultural University, Harbin 150030, China

**Keywords:** *in vitro* embryo culture, gastrulation, stem cell-derived embryo models, biomedical models

## Abstract

The post-implantation phase of mammalian development is crucial yet challenging to study due to ethical and technical constraints, particularly in humans. Recent revolutionary advances in extended *in vitro* culture systems for mammalian embryos now offer unprecedented windows into this developmental “black box”. This review synthesizes how these platforms, alongside stem cell-derived embryo models, are transforming our ability to model early human development in a dish. We detail the technological evolution from two-dimensional (2D) to three-dimensional (3D) cultures that support mouse, non-human primate, and human embryos through key stages of implantation and gastrulation, recapitulating events like lineage specification and axial patterning. Furthermore, we explore how these models serve as powerful tools for investigating the etiology of early pregnancy failure, screening for developmental toxicity of pharmaceuticals, and deciphering the molecular pathogenesis of birth defects. By bridging fundamental embryology with clinical and pharmacological applications, these innovative models herald a new era in biomedical research, holding significant promise for advancing reproductive medicine and regenerative strategies.

## 1. Introduction

Understanding post-implantation mammalian embryogenesis remains a central goal in developmental biology. This phase involves critical morphogenetic events and cell fate decisions that establish the foundational body plan. However, direct observation in utero, especially in humans, is hindered by ethical and practical limitations. Consequently, researchers have turned to model organisms and *in vitro* systems to bridge this knowledge gap [1,2].

Mammalian development begins with the zygote, a totipotent cell formed by gamete fusion [3]. The pre-implantation stages—from zygote to blastocyst—are highly conserved across species and involve zygotic genome activation (ZGA) [4,5], compaction, morula formation, and the segregation of the inner cell mass (ICM) and trophectoderm (TE) [6,7]. The TE facilitates blastocoel formation, leading to the mature blastocyst [8,9,10].

**Figure 1:**
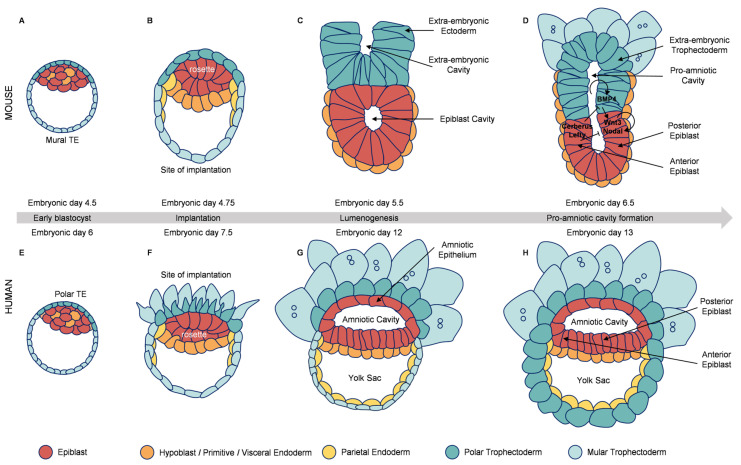
Key developmental events and species-specific differences during the peri-implantation period in mammals. Schematic comparing key developmental events in mouse (**A**–**D**) and human (**E**–**H**) embryos from the late blastocyst stage through early post-implantation. (**A**,**E**) Pre-implantation blastocysts show conserved lineage segregation (ICM/TE). (**B**,**F**) Post-attachment epiblast polarization and rosette formation. A key morphological divergence emerges upon implantation: mouse embryos form a cup-like egg-cylinder (**C**,**D**), while human embryos develop as a flat bilaminar disk (**G**,**H**). (**C**,**G**) Cavity expansion (pro-amniotic in mouse, amniotic in human). (**D**,**H**) Establishment of the species-specific body plan foundation, highlighting that conserved signaling centers (e.g., *BMP4*, *Nodal*) pattern the anterior–posterior axis despite the differing tissue geometry. The gray arrow indicates the developmental timeline (from early blastocyst to pro-amniotic cavity formation).

Upon hatching, the blastocyst implants into the uterus. The TE differentiates into polar and mural subtypes, while the ICM gives rise to the epiblast (Epi) and primitive endoderm (PE) [11,12,13,14] (Figure 1A,B,E,F). Post-implantation morphogenesis diverges significantly among species; for instance, mouse embryos form an egg-cylinder (Figure 1C), whereas human embryos develop as a bilaminar disk (Figure 1G) [15,16,17,18]. These differences underscore the limitations of relying solely on mouse models and highlight the need for direct human embryological studies.

The post-implantation Epi is essential for gastrulation and axis formation. In mice, signaling centers such as the extraembryonic ectoderm (ExE) and anterior visceral endoderm (AVE) regulate anterior–posterior patterning through factors like *BMP4*, *Nodal*, *Wnt3*, and antagonists including *Lefty1* and *Cer1* (Figure 1D,H) [19,20,21,22]. Similar mechanisms are conserved in non-human primates, suggesting shared patterning logic among primates [23,24].

Given these interspecies differences, direct study of human post-implantation development is indispensable. Recent advances in prolonged *in vitro* culture systems now enable the sustained development of mouse, non-human primate, and human embryos through key developmental stages. This review outlines the historical breakthroughs in these culture technologies, summarizes the methodologies, and discusses their implications for understanding human development and disease.

## 2. The Foundational Role of the Mouse Model

The successful establishment of *in vitro* culture systems for early mouse embryos represents a pivotal breakthrough in developmental biology. These systems have, for the first time, enabled the continuous development of mouse embryos from the initiation of implantation through to the gastrula stage ex utero [25,26,27,28]. This achievement systematically validates the feasibility that embryos can autonomously execute a series of complex developmental events—including blastocyst attachment, lineage segregation, and gastrulation—without direct support from the maternal uterus. This milestone not only confirms the high degree of intrinsic autonomy underlying post-implantation embryonic development but also provides an indispensable technical foundation for subsequent studies involving higher mammals and even human embryos [29].

The development of early mouse embryo *in vitro* culture systems has been a process of gradual optimization and successive breakthroughs. The evolution of these systems is clearly reflected in the coordinated refinement of key components—such as culture medium composition, gas conditions, and cultivation methods—which ultimately supported complete embryo development from implantation to gastrulation.

The pioneering work of Hsu in 1973 demonstrated that E3.5 blastocysts could develop to the early somite stage (equivalent to ~E8.5) over approximately 5 days using a sequential serum strategy in MEM medium, transitioning from fetal bovine serum (FBS) to human cord serum (HCS) on collagen-coated dishes under static conditions [30]. A significant leap was achieved by Chen and Hsu in 1982, who utilized CMRL-1066 medium supplemented with FBS, HCS, and rat serum (RS) [31]. By innovatively integrating static with agitation/roller culture, they extended the development of E3.5 blastocysts to an E9.5-equivalent stage (~10 days), observing a beating heart and blood circulation. Entering the 21st century, the focus shifted toward more defined culture conditions. Bedzhov et al. adopted Advanced DMEM/F12 supplemented with insulin-transferrin-selenium (ITS-X) and knockout serum replacement (KSR), significantly reducing the reliance on complex sera [29,32]. This system, often employing specialized substrates like ibiTreat μ-plates under static culture, robustly supported embryo development to the egg cylinder stage (equivalent to E6.5) within 2–5 days. The most extensive duration was recently reported by Aguilera-Castrejon et al. [15]. Using DMEM medium with FBS and RS under precisely regulated, stage-specific oxygen conditions, they implemented a critical culture regimen: static conditions from E5.5 to E8.5, followed by a mandatory switch to dynamic roller culture beyond E8.5. This approach enabled the continuous development of E5.5 embryos for 6 days, reaching E11 with 42 somite pairs (Table 1). The historical evolution of mouse embryo culture systems, summarized in Table 1, represents more than a technical checklist. It systematically unveils a core biological principle: the progressive and stage-specific autonomy of the post-implantation embryo. Early work established the feasibility of autonomy; subsequent refinements defined the biochemical and physical inputs required to manifest it through gastrulation and into organogenesis.

Collectively, these studies have established core principles for successful extended culture—including sequential nutritional support, dynamic gas regulation, and stage-specific physical stimulation. This methodological evolution has not only systematically validated the intrinsic autonomy of post-implantation mouse development but also provided an indispensable technical and conceptual framework for subsequent studies on non-human primate and human embryos.

## 3. The Bridge Function of Non-Human Primate Models

Building on the foundation laid by mouse studies, researchers have established robust *in vitro* culture systems for non-human primate (NHP) embryos, primarily using the cynomolgus monkey (*Macaca fascicularis*). These systems have successfully supported embryo development beyond early gastrulation, providing an indispensable and physiologically relevant model for inferring human embryonic development, especially beyond the ethical *in vitro* culture limit of 14 days.

The evolution of NHP embryo culture is marked by progressive optimization. Early attempts, such as those by Enders et al. in 1989, demonstrated the feasibility of culturing fertilized rhesus monkey ova to the blastocyst stage, though with limited post-implantation development [33]. A significant advancement came from Lopata et al. in 1995, who cultured marmoset blastocysts on Matrigel using MEM medium supplemented with 10% FBS, insulin, and transferrin, observing syncytiotrophoblast formation and amniotic cavity development over 4–6 days [34] (Table 2).

The field of primate embryology was revolutionized in 2019 by two seminal, independent studies that established robust long-term *in vitro* culture systems for cynomolgus monkey embryos, enabling development up to 20 days post-fertilization and thus surpassing early gastrulation [24,35] (Table 2). Ma and Niu et al. pioneered a system utilizing a DMEM/F12-based medium supplemented with FBS and KSR, culturing blastocysts on Matrigel under static conditions [23,24]. This approach successfully recapitulated pivotal developmental events, including bilaminar disk formation and primordial germ cell specification. Concurrently, Ma et al. achieved comparable milestones using a CMRL-1066-based medium enriched with a cocktail of FBS, KSR, and RS, thereby independently validating the feasibility of extended NHP embryo culture. The success of these and subsequent advanced systems hinges on a meticulously designed, sequential culture strategy that mirrors the dynamic *in vivo* microenvironment. The protocol typically initiates with an attachment phase, employing a medium such as DMEM/F12 fortified with 20% FBS, key hormones (β-estradiol, progesterone), and the critical addition of the ROCK inhibitor Y-27632 to enhance embryo survival by suppressing apoptosis during the stress of implantation *in vitro*. This medium supports the attachment of zona pellucida-free blastocysts to a Matrigel matrix. Following successful attachment, the culture transitions to a long-term development phase, often characterized by the use of a more defined medium where serum is largely replaced by 30% KSR. This shift reduces batch-to-batch variability and provides a stable environment conducive to the maintenance and subsequent differentiation of pluripotent epiblast cells. These optimized culture platforms have demonstrated an exceptional capacity to recapitulate key *in vivo* developmental processes. Morphological and molecular analyses confirm that *in vitro*-cultured NHP embryos faithfully undergo epiblast-hypoblast segregation, amniotic cavity and yolk sac cavitation, bilaminar disk formation, emergence of primordial germ cells, and ultimately, primitive streak formation and anterior–posterior axis establishment. The high conservation of these events’ timing and morphology between cynomolgus monkeys and humans underscores the system’s strong physiological relevance and significantly bolsters confidence in its predictive value for human development [24,35]. Beyond morphology, high-resolution single-cell transcriptomic and chromatin accessibility analyses conducted on these *in vitro* systems have systematically decoded the dynamic molecular landscape underlying lineage specification. These studies have delineated the transcriptional profiles of diverse cell lineages—including EPI, PE, TE, extra-embryonic mesenchyme, amniotic epithelium, and gastrulating cells—and identified central roles for signaling pathways such as WNT, TGF-β, and Nodal in driving lineage segregation, epithelial-to-mesenchymal transition, and gastrulation. Consequently, these models provide an unprecedented, detailed molecular framework for understanding the regulatory logic of early post-implantation primate development, for which human data remains ethically inaccessible beyond the 14-day limit.

A further significant leap was achieved in 2023 with the introduction of sophisticated three-dimensional (3D) culture systems, which dramatically extended the *in vitro* development window and enhanced morphological fidelity [36] (Table 2). Zhai et al. developed a prolonged *in vitro* culture (pIVC) system by embedding cynomolgus blastocysts in a 3D Matrigel matrix within U-bottom low-adhesion plates, using a modified DMEM/F12-based medium [36,37,38]. This system supported over 33% of embryos to develop to day 25, achieving advanced milestones including neurulation, neural tube closure, and the emergence of early motor neurons. In parallel, Gong et al. reported a ‘sandwich-like’ 3D system, culturing embryos between layers of Geltrex and Matrigel, which also robustly supported embryogenesis to day 25 with an efficiency exceeding 20%. This system captured late gastrulation events, yolk sac hematopoiesis, and the specification of lateral plate mesoderm, demonstrating a broader range of organogenesis-associated processes.

**Table 2:** Key Advances in Extended *In Vitro* Culture of Non-Human Primate Embryos.

Year and Investigator	Species	Key Technical/Contextual Advance	Max. Duration	Key Developmental Milestones Achieved	Key Biological Insight Enabled
Enders et al. (1989) [33]	Rhesus Monkey	Demonstrated feasibility of IVF and blastocyst culture in primates.	10–13 days	Blastocyst formation, limited attachment	Foundation: Confirmed basic primate pre-implantation development can be supported *in vitro*, paving the way for post-implantation studies.
Lopata et al. (1995) [34]	Marmoset	Use of Matrigel matrix to support post-attachment development.	11 days	Syncytiotrophoblast, Amniotic Cavity	Early morphogenesis: Showed that a 3D matrix could support initial trophoblast differentiation and amniogenesis in a primate model, key early post-implantation events.
Niu et al. (2019) [24]	Cynomolgus	Defined, serum-reduced system (DMEM/F12 + KSR) for long-term static culture.	20 days	Bilaminar Disk, Amnion, PGCs, Gastrulation	Primate-specific blueprint: Provided the first comprehensive model of primate bilaminar disk formation and primordial germ cell (PGC) specification ex utero, highlighting conserved signaling with rodents in a divergent morphology.
Ma et al. (2019) [23]	Cynomolgus	Alternative medium formulation (CMRL-1066 + sera) achieving similar long-term development.	20 days	Gastrulation, Three Germ Layers	Independent validation and gastrulation: Independently confirmed the autonomous capacity for primate gastrulation *in vitro*, reinforcing the reproducibility and robustness of the model system.
Zhai et al. (2023) [37]	Cynomolgus	Advanced 3D embedding in Matrigel (“pIVC” system).	25 days	Neurulation, Neural Tube, Early Organs	Extended self-organization: Demonstrated that primate embryos can autonomously progress to neurulation and early organogenesis without maternal input, revealing profound self-patterning potential beyond gastrulation.
Gong et al. (2023) [38]	Cynomolgus	“Sandwich” 3D culture (Geltrex/Matrigel).	25 days	Late Gastrulation, Yolk Sac Hematopoiesis	Modeling later events: Captured yolk sac-mediated hematopoiesis and lateral plate mesoderm specification, expanding the window for studying organogenesis-associated processes in a primate model.

Hormones typically include β-estradiol, progesterone, and N-acetyl-L-cysteine.

The successful establishment of long-term NHP embryo culture systems (summarized in Table 2) extends beyond a technical replication of mouse methods. It provides a critical validation bridge in three key dimensions: (1) These studies have demonstrated that primate embryos possess the intrinsic ability to undergo gastrulation and initiate neurulation *in vitro*. This not only confirms the conserved logic of developmental patterning but also recapitulates human-specific morphogenesis, such as the formation of the bilaminar disk. (2) They verified that the culture principles established in mice (such as sequential media formulations and 3D extracellular matrices) are also applicable to primates, thereby de-risks the translation of these protocols to human embryo studies. (3) By enabling the study of gastrulation in NHP, this system provides a physiologically relevant and ethically tractable model for investigating post-implantation developmental stages that extend beyond the current 14-day limit for human embryo culture. Consequently, it helps to refine and ethically ground the fundamental scientific questions that can be addressed in human embryology. Milestones in Human Embryo Culture

The *in vitro* culture of human embryos represents the frontier of developmental biology, enabling direct investigation of post-implantation events that are otherwise inaccessible. Guided by the ethical “14-day rule,” this field has progressed through refined culture methodologies, evolving from simple 2D systems to more physiologically relevant 3D platforms that better recapitulate the architecture and self-organizing principles of early human development.

### 3.1. Two-Dimensional Culture

Based on studies by Shahbazi et al. and Zhou et al., an *in vitro* culture system for human embryos has been established that successfully supports development from the blastocyst stage to day 14 through a defined sequential media strategy and standardized static culture conditions [17,35,39] (Table 3). The core of this system involves a stage-specific media transition: an initial attachment medium (IVC1), based on Advanced DMEM/F12 and supplemented with 20% fetal bovine serum along with key hormones (β-estradiol, progesterone, and often N-acetyl-L-cysteine) and additives (L-Glutamine, ITS-X Supplement and Penicillin/Streptomycin), is used to mimic the maternal microenvironment during the implantation window, promoting zona pellucida removal and initial attachment. This is followed by a long-term development medium (IVC2), in which serum is replaced with 30% KnockOut Serum Replacement to provide a more stable, chemically defined environment conducive to the maintenance of pluripotent epiblast cells and subsequent lineage specification. The entire culture process is carried out in Matrigel-coated plates under conditions of 37 °C, 5% CO_2_, and 21% O_2_, ensuring structured and functional development within a three-dimensional biosupport.

The operational procedure emphasizes precise timing and standardization. After removal of the zona pellucida, embryos are first cultured in IVC1 for approximately 48 h to achieve attachment. The medium is then switched to IVC2, with half-medium changes performed daily to dynamically meet the changing requirements of the embryo during attachment, lineage segregation, and pre-gastrulation stages. Notably, the study demonstrated that a 21% O_2_ environment is essential for sustaining epiblast survival and cellular polarization, whereas 5% O_2_ hypoxia leads to loss of pluripotency—a critical insight for optimizing gaseous parameters *in vitro*.

**Figure 2:**
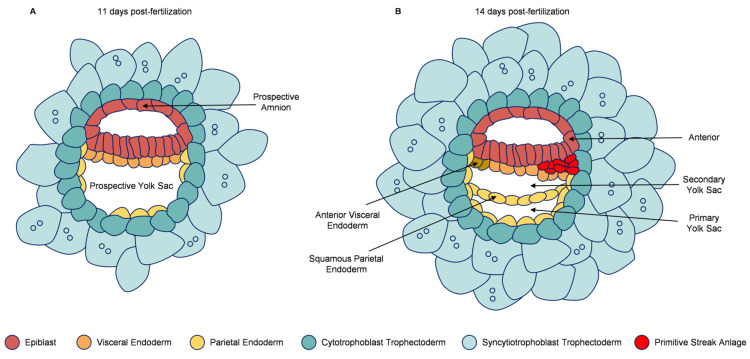
Comparative outcomes of 2D versus 3D *in vitro* culture systems for human embryos. (**A**) A representative image of a human blastocyst developing in a 3D culture system, forming a bilaminar disk with amniotic and yolk sac cavities. (**B**) Schematic comparing the developmental milestones achievable in 2D versus 3D *in vitro* culture. While 2D systems support basic lineage segregation and polarization, only 3D environments recapitulate advanced morphogenetic events, demonstrating that the human embryo possesses an intrinsic capability for autonomous axis formation and tissue compartmentalization in the absence of maternal tissues.

In summary, the establishment of 2D culture systems represented a pivotal first step, providing the initial proof-of-concept that key aspects of human post-implantation development can be recapitulated *in vitro* without maternal tissues. These systems successfully modeled fundamental developmental events—including implantation initiation, epiblast polarization, pro-amniotic cavity formation, and bilaminar disk establishment (Figure 2A). These pioneering platforms not only confirmed the remarkable self-organizing capacity of the human embryo but, more importantly, established the essential methodological framework—including sequential media strategies and defined matrix support—that directly enabled the subsequent development of more advanced 3D culture technologies.

### 3.2. Three-Dimensional Culture

Compared with traditional two-dimensional (2D) culture systems, the core innovation of the 3D system established by Li et al. lies in the synergistic optimization of both culture medium composition and extracellular matrix conditions [40] (Table 3). First, by supplementing the original IVC1 and IVC2 media with sodium lactate and sodium pyruvate to better mimic the physiological conditions of the fallopian tube and uterine fluids and support embryonic metabolic demands, along with the ROCK inhibitor Y27632 to suppress stress-induced cell death, the researchers developed modified media (mIVC1 and mIVC2), which significantly improved the embryo survival rate to 23.4%. Second, through systematic screening of different Matrigel concentrations as a 3D scaffold, 10% Matrigel was identified as the optimal condition for effectively promoting the formation and maintenance of self-organized 3D embryonic structures, including characteristic morphological features such as the amniotic cavity, bilaminar disk, and primary and secondary yolk sacs.

In terms of specific culture strategy, the study employed low-adhesion 96-well plates for single-embryo culture. On day 9, embryos were embedded in mIVC2 medium containing 10% Matrigel, followed by daily half-medium changes under stable environmental conditions (37.2 °C, 6% CO_2_, and saturated humidity). This system not only supported the morphological recapitulation of key developmental milestones such as anterior–posterior axis establishment and primitive streak anlage emergence, but also demonstrated—through single-cell transcriptomic sequencing and multilineage immunofluorescence staining (e.g., *OCT4*, *GATA6*, *CK7*, *T/Brachyury*)—that the resulting cell type differentiation and gene expression dynamics closely resembled those of *in vivo* embryos.

The 3D human embryo culture system has achieved several major breakthroughs in modeling early post-implantation development [41,42]. First, the 3D system significantly enhances the recapitulation of three-dimensional embryonic architecture and key morphogenetic events (Figure 2B). While embryos under 2D culture conditions attach and spread into flattened structures, the 3D environment successfully supports the establishment of complex configurations such as the bilaminar disk, pro-amniotic cavity, secondary yolk sac, and the segregation of amniotic epithelium from the epiblast [17,40,43]. Moreover, trophoblast cells in 3D culture differentiate into cytotrophoblast and syncytiotrophoblast subtypes, forming characteristic multinucleated structures and lacunae—features rarely observed in 2D conditions. Second, the 3D system substantially extends the duration of *in vitro* development and enhances embryonic self-organization. In 2D culture, embryonic development typically arrests around day 10 with limited proliferative capacity, whereas 3D-cultured embryos can develop through day 14 while maintaining continued proliferation across lineages and structural expansion [17,40,44]. Importantly, studies have revealed that even in the absence of maternal tissues, human embryos retain the autonomous capacity to accomplish key developmental milestones—including anterior–posterior axis patterning and primitive streak anlage formation—highlighting their intrinsic self-organizing potential. Finally, the 3D platform offers a more physiologically relevant system for investigating cellular behavior and developmental mechanisms [45]. Through single-cell transcriptomics and other high-resolution approaches, researchers can precisely dissect molecular pathways and gene regulatory networks underlying lineage specification—insights often unattainable in 2D systems due to structural incompleteness or developmental arrest.

In summary, the establishment of these defined human embryo culture systems has been instrumental in decoding the complex events of the first two weeks of human development. They have not only confirmed the remarkable self-organizing capacity of the human embryo in the absence of maternal tissues but have also set a gold standard for assessing the fidelity of stem cell-derived embryo models. The technological progression from 2D to 3D culture marks a pivotal shift towards achieving greater physiological relevance *in vitro*, thereby providing an indispensable, ethically constrained platform for exploring the fundamental principles of human life and the etiology of early pregnancy disorders.

## 4. From Embryos to Models: Stem Cell-Derived Embryo Models as a Complementary System

The study of early mammalian embryonic development, particularly the key events surrounding implantation, has long been hampered by the inaccessibility of *in vivo* models and the scarcity of human embryo materials [46]. Although *in vitro* embryo culture techniques have advanced, they remain limited in developmental duration and are not amenable to genetic manipulation [47]. In this context, the use of pluripotent stem cells to self-assemble into embryoids has emerged as a crucial complementary strategy for deciphering this developmental “black box” [48,49]. The field has evolved along a path from “partial mimicry” to “integrated reconstruction,” and from “mouse models” to “human systems,” with the core scientific goal of uncovering the self-organizing principles that drive early embryogenesis and lineage specification [50,51].

**Figure 3:**
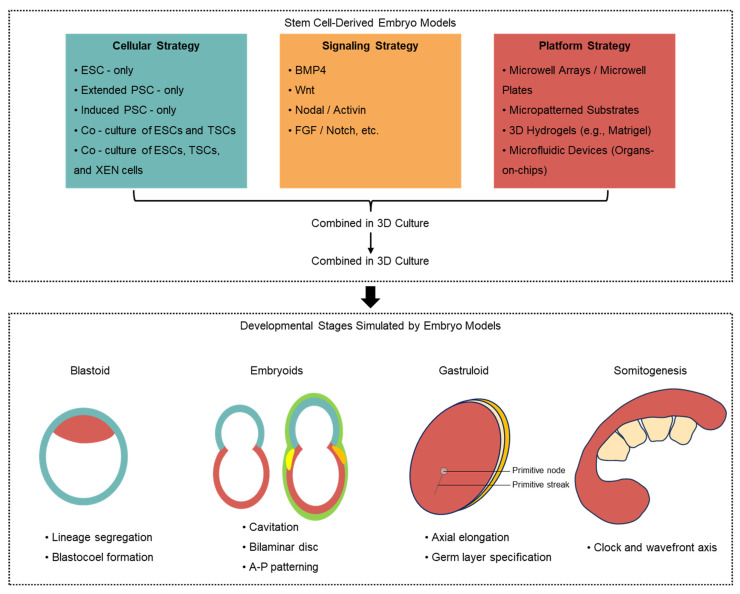
Engineering stem cell-based embryo models to dissect self-organization. (**Top**) The three core strategies for constructing embryo models are based on: (i) co-culturing distinct stem cell lineages (e.g., ESCs, TSCs, XEN cells) in 3D, (ii) modulating the biochemical environment with signaling factors (e.g., *BMP4*, *Wnt*, *Nodal*), and (iii) employing engineered biophysical platforms (e.g., microwell arrays, hydrogels, microfluidic devices). (**Bottom**) The developmental stages recapitulated by these models, ranging from blastoid to somitogenesis-like stages, demonstrating progressive structural, axial, and morphogenetic complexity.

The construction of these embryoids is conceptually framed as an engineering problem, relying on the synergistic integration of three core components: (i) the cellular building blocks, (ii) the biochemical environment, and (iii) the biophysical context (Figure 3).

Cellular Building Blocks: The choice of stem cells determines the intrinsic developmental potential of the model. Early breakthroughs demonstrated that mouse embryonic stem cells (ESCs) alone could form embryoid bodies and, under specific signaling cues, more advanced gastruloids that recapitulate aspects of axial patterning [52,53]. A significant leap came from incorporating extra-embryonic lineages [50]. The combination of ESCs with trophoblast stem cells (TSCs) gave rise to blastocyst-like structures (blastoids) [41,54,55], while the subsequent integration of extraembryonic endoderm (XEN) stem cells enabled the formation of post-implantation embryoids (e.g., ETS and ETX embryos) that mimic the embryonic-extraembryonic crosstalk essential for gastrulation [56,57,58].

Biochemical Environment (Signaling Factors): Precise control of the chemical milieu is crucial for guiding cell fate and morphogenesis. The homogeneous application of key morphogens is sufficient to trigger spontaneous self-organization and establish endogenous signaling gradients [59,60,61,62]. For instance, Wnt agonists (e.g., CHIR99021) can induce primitive streak-like patterning in gastruloids. Similarly, BMP4 stimulation in 2D micropatterned hESC colonies is capable of triggering germ layer specification [61]. This demonstrates that external biochemical cues can robustly activate the intrinsic self-patterning capabilities of stem cell aggregates.

Biophysical Context (Engineered Platforms): The physical microenvironment provided by bioengineered platforms is a fundamental determinant of self-organization. Microwells facilitate the standardized aggregation of stem cells [63,64]. Micropatterned substrates geometrically confine cells in 2D, enabling quantitative studies of signaling and mechanics [65,66]. Three-dimensional hydrogels like Matrigel provide essential mechanical support and biochemical cues that promote the formation of complex architectures, such as the pro-amniotic cavity and somite-like structures [67,68]. Emerging microfluidic devices offer dynamic control over the culture environment, including the spatiotemporal presentation of morphogen gradients, further enhancing the physiological relevance of these models [69].

Building on the solid foundation in mouse studies, research on human embryo models has surged. Multiple teams have independently reported the generation of human blastoids from pluripotent stem cells [70,71]. These models closely resemble human blastocysts in morphology and transcriptome and can initiate peri-implantation-like development. Like their mouse counterparts, human blastoids and gastruloids offer significant advantages in scalability and genetic tractability, substantially compensating for the ethical and practical limitations of human embryo research.

However, it is crucial to recognize that these models are not equivalent to real embryos. Current embryoids often exhibit deviations in lineage proportions, spatial organization, and developmental potential [50]. They cannot progress beyond the primitive streak stage and are incapable of forming viable fetuses. Despite these limitations, embryo models and genuine embryo culture technologies have established a relationship of mutual validation and complementarity: embryo data provide a gold standard for evaluating models, while the models offer a highly intervenable system for reverse-validating developmental hypotheses. Together, they advance our understanding of the earliest stages of mammalian life.

## 5. Conclusions and Perspectives

Collectively, the establishment of robust *in vitro* embryo culture systems and the emergence of stem cell-derived embryo models have fundamentally transformed our approach to studying post-implantation mammalian development. By providing complementary, accessible, and ethically tractable windows into the once-inaccessible “black box” of early embryogenesis, these technologies have bridged a critical knowledge gap. As a comparative analysis of key operational parameters across species illustrates (Table 4), these systems now offer a more nuanced bridge between mouse models and human development. This paradigm shift not only deepens our fundamental understanding of self-organization, lineage specification, and morphogenesis but also unlocks immense potential for translational applications in modeling pregnancy disorders, screening teratogens, and informing regenerative strategies [46,49,72]. 

Significant progress has been made in the *in vitro* culture of mammalian embryos and the development of embryoids, enabling researchers to systematically observe and analyze key developmental events around the period of implantation [47,73]. However, current technological systems still exhibit notable limitations [15]. Firstly, neither directly cultured embryos nor stem cell-derived embryoid structures can currently be sustained *in vitro* through the early stages of organogenesis [51]. Secondly, *in vitro*-cultured embryos still display structural and functional disparities compared to their *in vivo* counterparts, particularly in overall size, vascular network formation, and the development of functional extra-embryonic tissues (such as the placenta), which restricts their utility in modeling complete embryonic development [74].

Looking forward, advancements in this field will rely on the integration of multidisciplinary technologies. On one hand, the construction of more complex “synthetic embryo” models could be achieved by combining organoids, organ-on-a-chip systems, and AI-assisted dynamic imaging and analysis, enabling real-time monitoring and precise regulation of the culture process [75,76,77]. On the other hand, leveraging such highly controllable platforms will facilitate the systematic dissection of gene function during embryogenesis and allow for the assessment of environmental factors—such as exogenous toxins and nutritional metabolism—in early embryonic development [41,47]. This will provide new insights into the mechanisms underlying birth defects and pregnancy loss.

The progression of this foundational research will not only deepen our understanding of early human developmental programming but also provide a theoretical basis for stem cell-based medicine and regenerative strategies. Furthermore, breakthroughs in understanding the principles of embryonic self-organization and improving techniques for ex utero maintenance will establish critical theoretical and technical foundations for future developments, such as “artificial womb” systems [78,79]. Ultimately, these efforts will propel developmental biology and reproductive medicine into a new era.

## Figures and Tables

**Table 1:** Key Advances in Extended *In Vitro* Culture of Mouse Embryos.

Year and Investigator	Max. Duration and Endpoint	Key Technical Innovation	Key Biological Insight Enabled
Hsu et al. (1973) [30]	~9 days to early somite (~E8.5)	Sequential serum strategy (FBS → HCS); static culture on collagen.	Proof of concept: Post-implantation mouse embryos possess substantial autonomous developmental capacity ex utero, independent of continuous maternal support
Chen et al. (1982) [31]	~10 days to E9.5-equivalent	Integration of multiple sera (FBS, HCS, RS); transition from static to agitated/roller culture.	Extended autonomy: Embryos can self-direct early organogenesis (cardiogenesis) *in vitro*, revealing the sufficiency of defined temporal cues for complex morphogenesis.
Bedzhov et al. (2014) [29,32]	~2–5 days to Egg cylinder (~E6.5)	Shift to defined medium (ITS-X, KSR); use of optical-grade substrates for imaging.	Mechanistic observation: Enabled real-time, high-resolution study of post-implantation lineage segregation and epiblast polarization, highlighting self-organizing properties.
Aguilera et al. (2021) [15]	6 days from E5.5 to E11 (with 42 somite pairs)	Stage-specific protocol: static (pre-E8.5) to dynamic roller culture (post-E8.5); precise gas regulation.	Late-stage self-organization: Demonstrated that embryos can autonomously progress through neurulation and somitogenesis, establishing the critical requirement for dynamic physical stimulation for mid-to-late stage development.

**Table 3:** Comparative Analysis and Biological Insights from 2D versus 3D *In Vitro* Culture of Human Embryos.

Feature/System Aspect	Two-Dimensional Culture System	Three-Dimensional Culture System	Key Biological Insight Enabled by the Comparison
Core Design Principle	Monolayer adhesion on coated surface; medium-centric optimization.	Embryo embedded within a 3D extracellular matrix (e.g., Matrigel); synergistic medium-matrix support.	The biophysical context (3D matrix) is not merely a scaffold but an active instructor of morphogenesis, essential for establishing correct tissue architecture and mechanical cues absent in 2D.
Max. Culture Duration	~14 days (adheres to ethical limit, but development often arrests earlier ~day 10).	~14 days (adheres to ethical limit, with sustained development through day 14).	Human embryos possess an intrinsic program capable of driving development through the second week *in vitro*, but its full execution requires a permissive 3D microenvironment.
Key Developmental Milestones Achieved	Basic lineage segregation (EPI, PE, TE). Epithelial polarization. Pro-amniotic cavity initiation. Bilaminar disk formation (flattened).	Proper amniotic epithelium-EPI segregation and basement membrane formation. Secondary yolk sac (SYS) development. Anterior–posterior (A-P) axis specification. Primitive streak anlage (PSA) emergence. Trophoblast subtype differentiation (CTB, STB, EVT).	The self-organizing potential of the human embryo is tiered. Two-dimensional systems reveal basic cell fate decisions, while 3D systems unlock the capacity for autonomous axis formation, tissue compartmentalization, and complex extra-embryonic tissue patterning, mirroring key *in vivo* events.
Structural and Morphological Fidelity	Embryo flattens; lacks true 3D architecture and proper tissue boundaries.	Recapitulates *in vivo*-like 3D spatial organization (bilaminar disk, cavities, layered tissues).	Authentic human morphogenesis is inherently three-dimensional; 2D culture imposes geometric constraints that disrupt the self-organization of complex body plans.
Utility for Disease and Toxicity Modeling	Limited to early lineage defects; poor model for structural birth defects.	Superior for modeling dysmorphogenesis (e.g., axis patterning errors) and assessing compound effects on integrated tissue development.	Three-dimensional systems provide a more physiologically relevant and predictive platform for investigating the etiology of early pregnancy failure and the developmental toxicity of pharmaceuticals, bridging a critical gap between cell-based assays and *in vivo* studies.

**Table 4:** Comparison of Key Parameters of Embryo *In Vitro* Culture Systems Across Species.

Feature	Mouse	Cynomolgus Monkey	Human (2D)	Human (3D)
Starting Stage	E3.5 Blastocyst	D7-8 Blastocyst	D5-6 Blastocyst	D5-6 Blastocyst
Max. Culture Duration	~6 days (to ~E11)	~20 days	~14 days	~14 days
Basal Medium	DMEM, Advanced DMEM/F12	DMEM/F12	Adv. DMEM/F12	mIVC1/mIVC2
Key Supplements	FBS, Rat Serum	FBS, KSR, Y-27632	FBS, KSR	KSR, Lactate/Pyruvate Y-27632
Matrix	Not Specified	Matrigel	Matrigel	10% Matrigel (3D hydrogel)
Gas Environment	Stage-specific O_2_	5% CO_2_	5% CO_2_, 21% O_2_	6% CO_2_
Key Developmental Milestones Achieved	Early organogenesis	Gastrulation, Early neural plate	Bilaminar disk, Amniotic cavity formation	A-P axis specification, Primitive streak anlage

## Data Availability

No new data were created or analyzed in this study. Data sharing is not applicable to this article.
